# A Novel Method of Vein Detection with the Use of Digital Image Correlation

**DOI:** 10.3390/e23040401

**Published:** 2021-03-28

**Authors:** Zbigniew Lutowski, Sławomir Bujnowski, Beata Marciniak, Sylwester Kloska, Anna Marciniak, Piotr Lech

**Affiliations:** 1 Faculty of Telecommunications, Computer Science and Electrical Engineering, UTP University of Science and Technology, 85-796 Bydgoszcz, Poland; slawomir.bujnowski@utp.edu.pl (S.B.); beata.marciniak@utp.edu.pl (B.M.); 2 Faculty of Medicine Ludwik Rydygier Collegium Medicum in Bydgoszcz, Nicolaus Copernicus University in Torun, 85-067 Bydgoszcz, Poland; mkloska93@gmail.com (S.K.); marciniak.anna1993@gmail.com (A.M.); 3 Department of Signal Processing and Multimedia Engineering, West Pomeranian University of Technology, 70-313 Szczecin, Poland; Piotr.Lech@zut.edu.pl

**Keywords:** vein detection, digital image processing, correlation, displacement measurement

## Abstract

Digital image correlation may be useful in many different fields of science, one of which is medicine. In this paper, the authors present the results of research aimed at detecting skin micro-shifts caused by pulsation of the veins. A novel technique using digital image correlation (DIC) and filtering the resulting shifts map to detect pulsating veins was proposed. After applying the proposed method, the veins in the forearm were visualized. The proposed technique may be used in the diagnosis of venous stenosis and may also contribute to reducing the number of adverse events during blood collection. The great advantage of the proposed method is the lack of the need to have specialized equipment, only a typical mobile phone camera is needed to perform the test.

## 1. Introduction

Measurement of displacements may be useful in many various fields. Because it is based on point-wise measurements, they offer solutions to relatively simple problems. One of the uses of the method could be in the medical field. The new application of digital image correlation (DIC), proposed in this paper, is to detect micro-shifts in the skin caused by pulsation of the underlying veins. The most important methods currently used for precise vein imaging are—Magnetic Resonance Angiography (MRA) methods, hyperspectral imaging methods (data captured in the range 900–1700 nm) and ultrasound imaging methods. However, mainly due to cost reasons, near infrared (NIR) with a wavelength from 740 nm to 760 nm imaging is the most popular method [1]. Use of vein finders minimalizes the risk of pre-analytical error, reduces potential pain of patient and is fast to perform. This method is based on the fact that hemoglobin absorbs the light emitted by diodes. Hemoglobin is composed of protein and iron containing compounds. The main function of hemoglobin is to transport blood rich in oxygen (O${}_{2}$) from lungs to all tissues of the body and blood rich in carbon dioxide (CO${}_{2}$) in the opposite direction [2]. It is also worth to mention that blood rich in oxygen will be transparent for NIR and will not visualize arteries. With the help of vein detector, it is possible to observe veins under the skin, as it appears in darker colors than background.

Medical diagnostics play a key role in the process of disease recognition. To provide the patient with the best quality medical care, it is important to plan the diagnostic procedure properly, starting with the sample. Blood is an extremely important source of information about the patient; therefore, it is most often used in diagnostic tests as a sample. Blood collection is an invasive method, so it is extremely important to choose veins accurately, so the patient experiences the least discomfort during the procedure. To obtain reliable information about a patient, samples should be taken in an appropriate manner, in order to reflect actual state of the patient. Many previous studies reported that there is a high percentage of error in blood sample collection. That is why it is extremely important to ensure proper preparation of the pre-analytical phase [3,4]. Peripheral venous access is very frequently performed procedure in hospitals. There are over 1 billion venipunctures performed every day, to obtain blood sample for testing. This procedure may be time-consuming and difficult in various groups of patients, such as neonates, children, obese patients, or patients after difficult, long treatment for example, chemotherapy. Venipuncture involves piercing a vein with a needle that makes it possible to draw it. This procedure can lead to patient stress, pain, and other unwanted reactions. Failures during blood collection can cause various types of complications, including thrombosis [5,6]. Such events can lead to court hearings in extreme cases because patients can hold people responsible for health damage. To locate a vein, a compression band and a gentle knock are usually used around the elbow joint. Locating vein is not easy due to various factors such as skin color, obesity, and dehydration of patients.

First attempts to use vein detectors were back in early 2000 [7]. A device, the Luminetx VeinViewer (LVV), produced by Luminetx Corp was tested. The aim of the work was to test whether LVV would be helpful in detecting feeder veins in various conditions and situations, especially in patients with telangiectasia. Device allowed localization of bigger number of veins than naked eye would, because feeder veins are too deep. Treating this disease was reliant on experience of physician, which is not a pleasant situation. LVV was compared to another device and showed a higher resolution and sensitivity. LVV proved to be an excellent device, however, the research was conducted on a relatively small group of 23 patients.

Along with globally increasing health awareness, the authors of [8] had the idea of developing an application that would allow mobile devices, such as smartphones or tablets, to act as vein detectors with the use of NIR. Considering the importance of portable devices in everyday life, it had a good chance of being successful. Nevertheless, it should be considered that the use of devices such as telephones is not a fairly reliable reference point from a medical point of view. Most applications that are now used to take care of health have not been tested for their effectiveness and reliable value of the results obtained. During their work they used vein detectors available on the market and had valuable clinical reviews. High cost and efficacy of studied vein detectors were not always as good as would be required. There were also several other problems with vein detectors and their evaluation. Most of the research was performed on small, homogenous groups which did not allow them to draw clear conclusions in the context of a larger, diverse population. Cost-effectiveness is one of the biggest problems for vein detectors. Those devices are not cheap themselves. They may even cost a few hundred dollars and they require trained personnel to use them. Preparations and training are also time consuming and cost money.

On the contrary, the authors of [9] created a device characterized by high-intensity and low-leak light source, near-infrared CMOS camera and a small and light one-eye head mounted display. With the use of these technologies, they could visualize veins at a depth of 67 mm. This allowed the performance of several venous interventions with a low chance of failure.

The authors of [10] also developed their version of vein detectors with the use of NIR. They used a CCD camera but were obliged to eliminate the cut-off filter to gain access to the infrared part of radiation spectrum. Source of light used to develop their detector were Infrared LEDs. Obtained images in resolution of 640 × 480 were then transferred to computer. Images were then processed with the use of several algorithms and programs, including LabVIEW. Proposed system managed to obtain vein images; however, they were not at the highest quality and indicated the need of future improvements in both software and hardware. Authors also pointed out that the vein detecting systems in general have difficulties with vein detection in people with many tattoos that blocked the transmission of light.

Despite many advantages, vein detectors also have disadvantages. One of which is the cost of devices and their portability. These facts can encourage researchers to continue their work to improve the devices and create reliable cost-effective and efficient vein finders for medical applications. It is also an area where roads of medics and biomedical engineers may cross. The concept of vein finders may seem to be simple, however, there are a lot of factors which hinder the task of creating the best one—to combine high quality devices with attractive price. The growth of interest in vein detectors led various producers to create their own devices.

Most of the vein detection methods based on image processing algorithms relate to biometrics. The use of the finger venous system due to its individual uniqueness. Biometric security features are much more effective than passwords or Personal Identification Numbers (PINs). Therefore, the authors of [11] proposed a method recognizing the venous pattern of the finger. The authors used algorithm based on gradient correlation. The proposed method consists in obtaining a high-quality image, which is then processed and stored in the database. The result of such operations is then compared with the pattern. Based on the arrangement of the finger veins, access can therefore be allocated or denied.

The authors of [12] used infrared images to detect finger veins. The method they proposed used the Gabor filter and the SIFT feature. It is characterized by a low error rate (below 0.5%). However, this method only focuses on the detection of the finger veins.

This is why the field needs to be more deeply explored and novel solutions need to be proposed. Most of the known solutions are based on the use of NIR, which automatically makes it necessary to have an illuminator and a camera working with such a wavelength of light. Looking for the cheapest solution that does not require any additional hardware components and is easily accessible to as many potential users as possible, our team decided to use a technique unique in this field—the correlation of images obtained with a camera of a typical mobile phone. The main idea of the method is based on the comparison of two images taken within a small amount of time, to capture skin deformation caused by blood flow. It is possible to increase the resolution of this method with the use of sub-pixel information calculations. There are many algorithms that allow this kind of calculation. One of the methods used for many years is the basic theory of digital image correlation (DIC), which enables the determination of image motion, distortions, or cracks. The theoretical accuracy of sub-pixel methods is well known; however, little is known about its real accuracy [13]. The goal of the research is to locate veins with the use of a camera directed at the examined limb. This allows observation of skin pulsations during blood flow through the veins. However, we should take into consideration the fact that it is impossible to keep a limb perfectly immobilized—the human body naturally shakes, which influences the quality of the obtained pictures. Regardless, it is possible to indicate pictures on which there are visible veins.

## 2. Methodology

### 2.1. Digital Image Correlation

**Figure 1:**
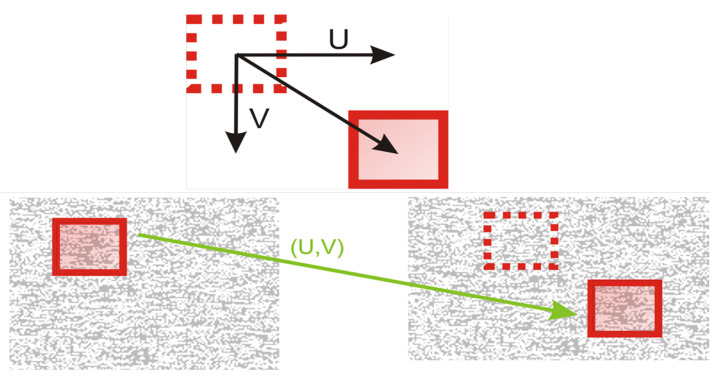
The principle of the digital image correlation (DIC) method.

The technique of digital image correlation is used for measuring displacements of textured objects. One image from the series is selected and it represents a reference picture for all subsequent analyses. Then it is divided into small rectangular regions (called subsets) containing N × N pixels (Figure 1). Subset size depends on the size of the random pattern, but also on its quality. Position of subsets is tracked from the reference picture to all other pictures from the measurement series. Cross-correlation coefficient calculation is used to search subsets positions. The displacement vectors U and V are calculated for every subset (Figure 1). Subpixel accuracy is achieved with the use of advanced interpolation methods, including Newton-Raphson method. This method is used to increase the accuracy of tracking, but also to speed up the process. At the beginning, it had extremely big computational costs, however, in recent years, scientists have been working on improving this algorithm, by removing its unnecessary parts. Thanks to their endeavors Newton-Raphson algorithm has become faster, and at the same time requires less computing power. This approach is a gold standard for detection of subpixel movement. The use of the Newton-Raphson algorithm during analysis improves the efficiency of the entire process. Set of displacement maps is obtained as a result, and therefore it can be used for deformation maps computation [14,15].

The DIC algorithm uses the correlation function to search for its maximum value. There are two different correlation criteria, which are used for the initial guess finding and its subsequent refinement. By computing at integer locations, the normalized cross correlation (NCC) (1) the initial guess is found.

(1) $$C_{cc}=\frac{\sum_{(i,j)\in S}\left(f\left({\tilde{x}}_{refi},{\tilde{y}}_{refj}\right)-f_{m}\right)\left(g\left({\tilde{x}}_{curi},{\tilde{y}}_{curj}\right)-g_{m}\right)}{\sqrt{\sum_{(i,j)\in S}}{\left[f\left({\tilde{x}}_{refi},{\tilde{y}}_{refj}\right)-f_{m}\right]}^{2}{\left[g\left({\tilde{x}}_{curi},{\tilde{y}}_{curj}\right)-g_{m}\right]}^{2}},$$

where *f* and *g* are respectively the reference and current image grayscale intensity functions at a specified location (x,y). Functions $f_{m}$ (2) and $g_{m}$ (3) correspond to the mean grayscale values of reference and current subset.

(2) $$f_{m}=\frac{\sum_{(i,j)\in S}(f\left({\tilde{x}}_{refi},{\tilde{y}}_{refj}\right)}{n(S)}$$

(3) $$g_{m}=\frac{\sum_{(i,j)\in S}(g\left({\tilde{x}}_{refi},{\tilde{y}}_{refj}\right)}{n(S)},$$

where n(S) is the number of data points in subset *S*. The initial guess yields *u* and *v* with integer (pixel) accuracy. To refine these results with sub-pixel resolution the next step is to use a nonlinear optimizer by finding the minimum of Equation (4).

(4) $$C_{LS}=\sum_{(i,j)\in S}{\left[\frac{f\left({\tilde{x}}_{refi},{\tilde{y}}_{refj}\right)-f_{m}}{\sqrt{\sum_{(i,j)\in S}[f\left({\tilde{x}}_{refi},{\tilde{y}}_{refj}\right)}{-fm]}^{2}}-\frac{g\left({\tilde{x}}_{refi},{\tilde{y}}_{refj}\right)-g_{m}}{\sqrt{\sum_{(i,j)\in S}[g\left({\tilde{x}}_{refi},{\tilde{y}}_{refj}\right)-g_{m}{]}^{2}}}\right]}^{2}.$$

In conclusion, in our research we performed the calculation of displacement maps by computing the NCC with sub-pixel accuracy obtained by applying the Newton-Raphson algorithm. At a later stage of the research, it turned out that it was necessary to develop additional methods of filtering the results, which were proposed in the section ’Methodology improvements and results’.

### 2.2. Data and Algorithm Parameters

**Figure 2:**
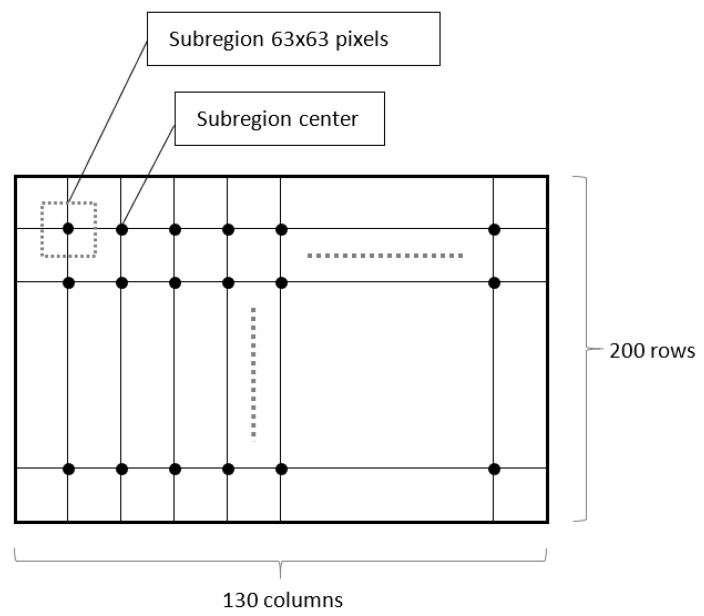
Grid of analyzed points.

The research work was carried out on data obtained with an ordinary mobile phone camera (Samsung Galaxy S10). Movies were recorded with 1920 × 1080 pixels resolution and 30 frames per second (FPS) speed. The resulting movie with a 23 s duration consisted of 675 frames (pictures). Since the physical dimensions of the area observed by the camera were 100 mm × 56 mm, a single pixel corresponded to a square with a side of 52 µm. The first frame was set as a base frame (BF) for the DIC algorithm, so the shifts were always calculated between BF and subsequent movie frames. To limit the calculation time, the area of interest has been defined as a grid designating points for which shifts were calculated. The grid consisted of 130 columns and 200 rows spaced 4 picture pixels apart (Figure 2). Each intersection of a column and a row marked a single pixel in the image for which the shift was calculated, resulting in total 26,000 points calculation for each pair of BF and *n*-th frame.

The use of DIC method for single pixel displacement calculation requires an analysis of its neighborhood. This neighborhood was defined as a square-shaped area (subregion) of 63 pixels side size.

## 3. Methodology Improvements and Results

**Figure 3:**
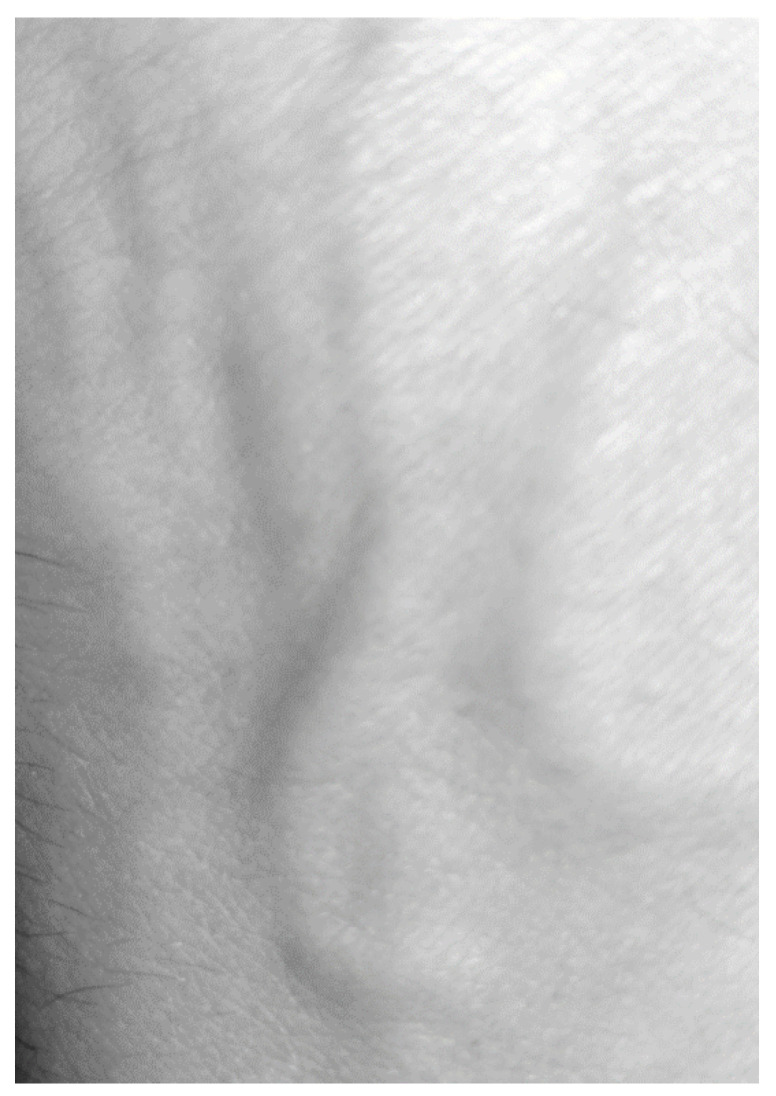
Base frame—No. 1 from a sequence of 675 frames (30 fps).

**Figure 4:**
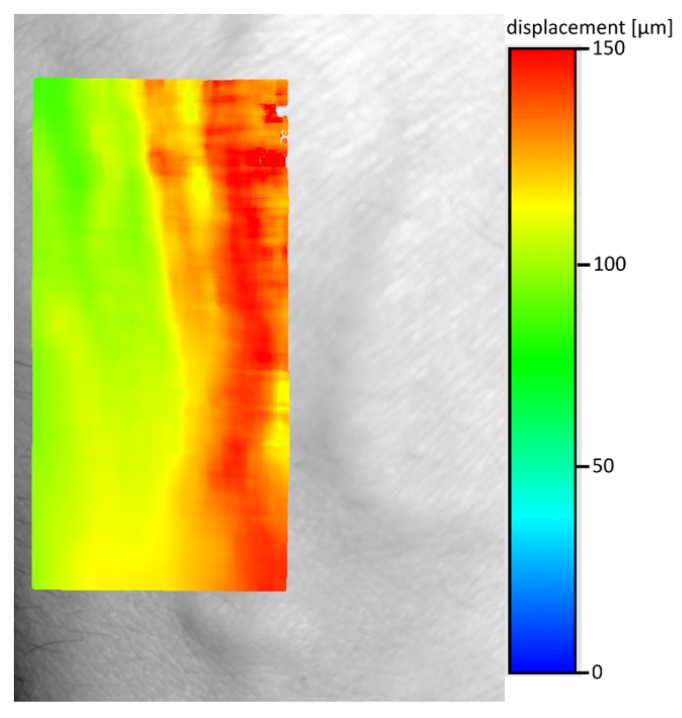
Shift between frames 1 and 206 with removal of the component designated by the area without visible veins of 100 × 300 pixels (the same value subtracted from all shifts).

The direct use of the methodology described in the previous chapter gave completely illegible shift maps. This was due to the fact that the limb vibrations were greater several orders of magnitude than the skin movements caused by the change in pressure during the blood flow through the blood vessel (Figure 3). Therefore, the resulting movements map was filtered using average shift calculated from 100 × 300 pixel area without visible veins. Then the calculated vector was subtracted from the shift map (Figure 4). The shifts maps on Figures 4, 6 and 8 are colored according to the absolute shift value, where the minimum shift value is blue and the maximum shift is red. Due to the poor visibility of the vessels at the edges of the hands, filtering using one, fixed shift vector turned out to be insufficient.

**Figure 5:**
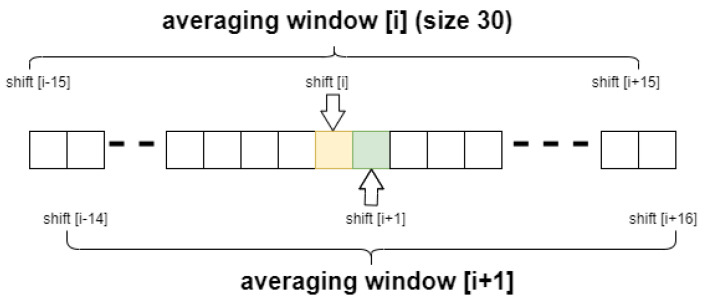
Schema of moving average calculation for exemplary *i* and *i*+1 shift.

**Figure 6:**
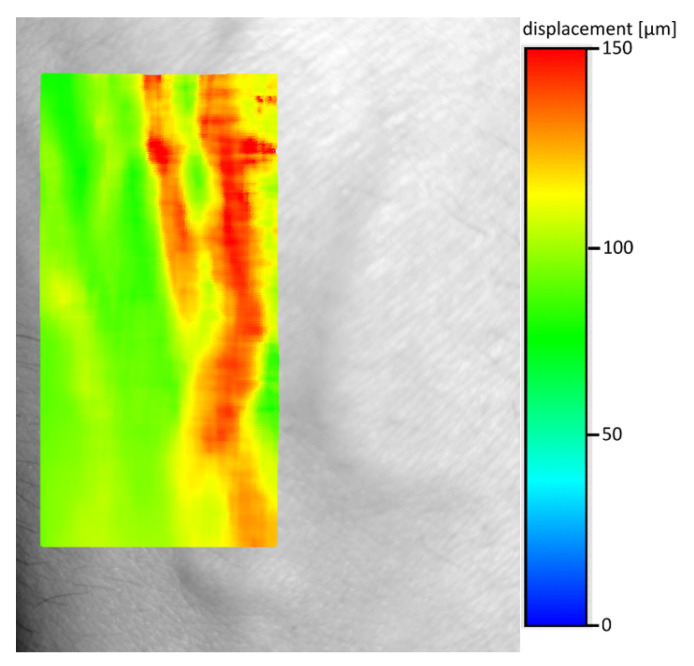
Shift between frames 1 and 206 with the removal of the component determined by a moving window (moving average) with a size of 30 pixels (15 on each side) (the window includes a single line).

The next improvement was to use the subtraction of the average calculated not once for the entire shift map, but the average determined by a sliding window, as shown on Figure 5. From each point in the shift map, the local average was subtracted. This local average was calculated from 30 adjacent shift points (15 from left and 15 from right), so each point in shift map was corrected by its own local average value. The resulting shifts map is shown on Figure 6. Comparison of Figure 4 and Figure 6 indicates better separation of vein edges in Figure 6. The optimal size of the averaging window, in terms of filtration, depends on the ratio of the distance between individual points of the offset map to the expected size of the vein. Thanks to the use of the window mechanism to determine the local mean, the effect of automatic adjustment of the algorithm to the non-uniform vector of the entire hand shift was achieved. These heterogeneities result from both the complicated nature of forehand vibrations and the distortions introduced by its curvature. Too small averaging window will “blur” the most interesting information about the location of the edges of the veins. On the other hand, too large will not consider the local changes in the optical shift of the entire hand area. The proposed value was selected experimentally.

**Figure 7:**
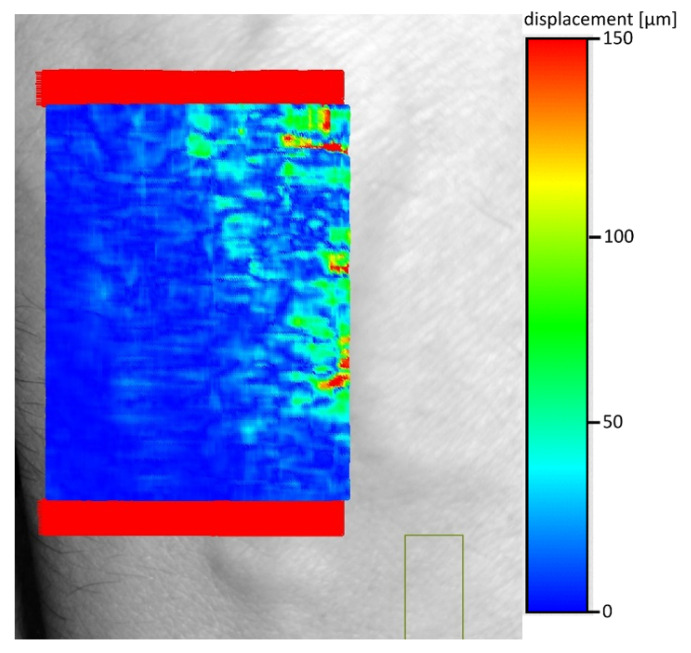
Detection of veins and arteries, image rotated 90 degrees (moving average determined from rows is not effective).

**Figure 8:**
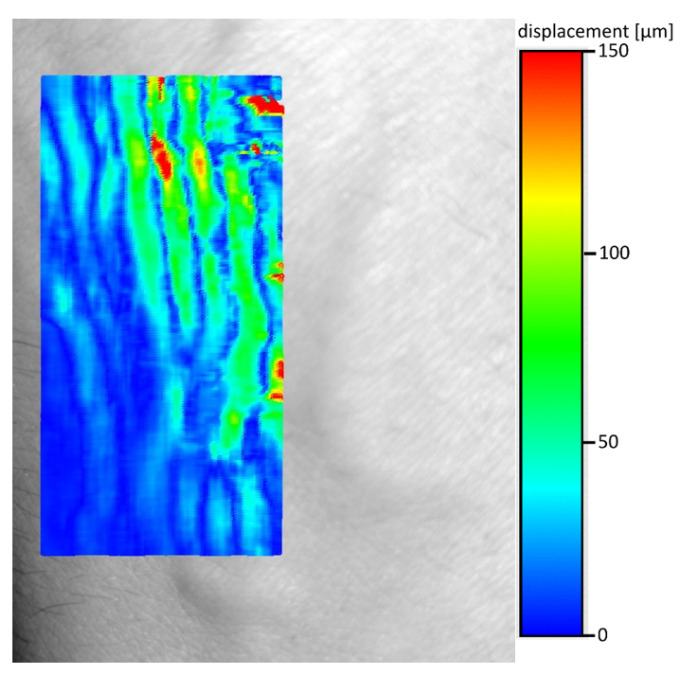
Shift between frames 1 and 423, moving average, visible edges of veins and arteries.

Because the averaging window is determined only based on a single row of shifts, the presented approach will not work for veins running parallel to the x-axis of the image (Figure 7). Since we focused on analyzing the images of the arm, we considered the above disadvantage to be insignificant at this stage of the research, but in the case of an unpredictable direction of the veins, it would probably be more appropriate to choose a filtering window with the size of not a single line, but the entire square area around the analyzed point. The resulting shifts map shows one more interesting effect—as it is clearly visible on Figure 8—map shows only edges of veins.

**Figure 9:**
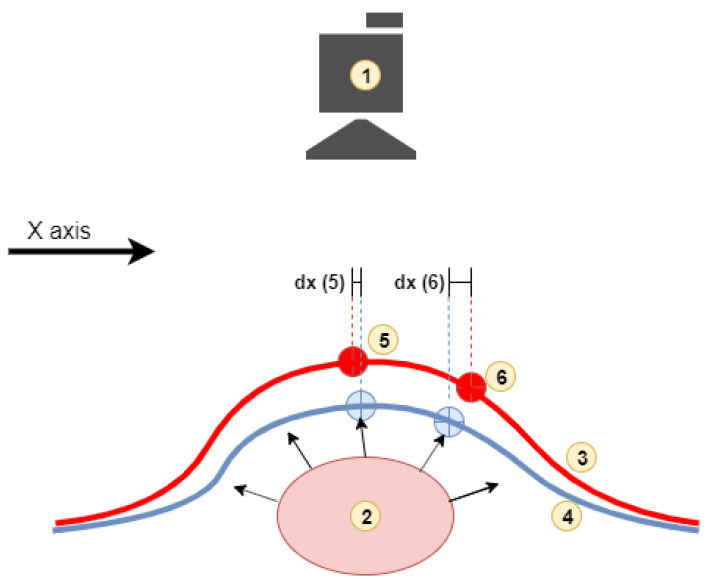
Explaining the mechanism causing visualization of the edges of the arteries (1—camera, 2—artery, 3—skin surface in the max pressure phase, 4—skin surface in the pressure min phase, 5 and 6 selected two points on the skin and their position in the maximum/minimum phase).

The vein edge detection mechanism causing visualization of two edges per single vein is explained in Figure 9. The method detects only shifts in axes parallel to the image plane, so the movement of point 6 has greater axis *X* delta value than the movement of point 5.

**Figure 10:**
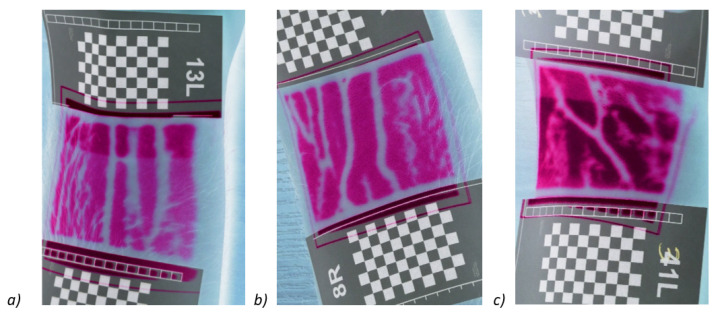
Negatives of the reference images (better readability when applied). (**a**) 13L, (**b**) 8R, (**c**) 31L describes the number of participant and which hand was tested, L—left, R—right.

In the next step, we tested the presented method on a group of 31 people. Veins were detected in each of the participants using the proposed method on both hands (Figure 10). The images of veins obtained with the proposed method were compared with the images obtained with the ‘Infrared vein finder Medcaptain NAVI-60’ device. Some examples of vein detection in one randomly selected participant is described below.

**Figure 11:**
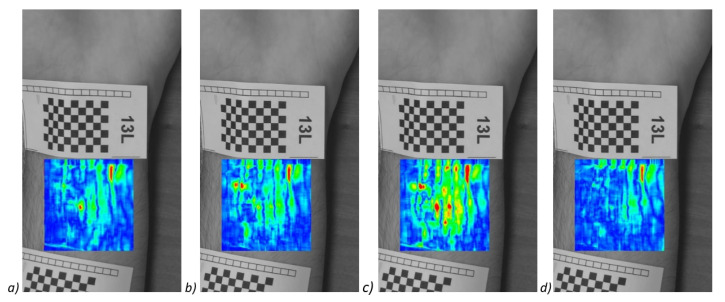
Exemplary images that makes up to the Figure 12—shift maps for frames #35,78,101,119 (**a**–**d**) of 120.

**Figure 12:**
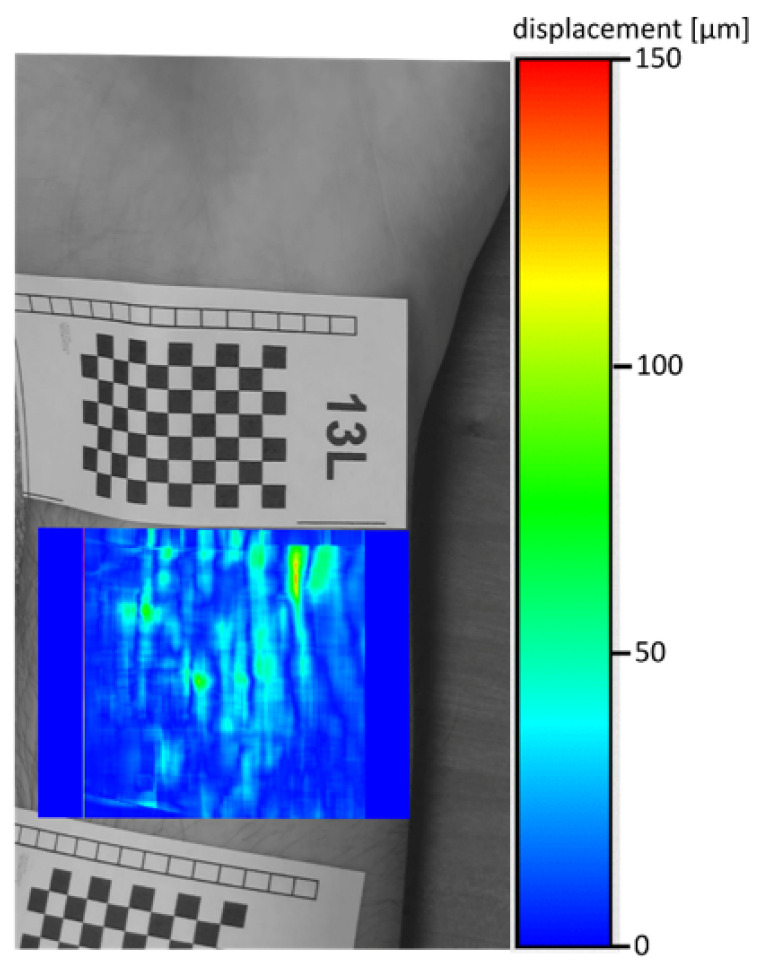
Cumulative shifts for 13L participant.

Presented method shows the image of the pulsation of the veins at a selected moment in time, in order to obtain a complete image of the veins, the frames of shifts from all time moments had to be summed up into one. Examples of images from different time moments are shown in Figure 11, which shows the shift maps making up the Figure 12.

**Figure 13:**
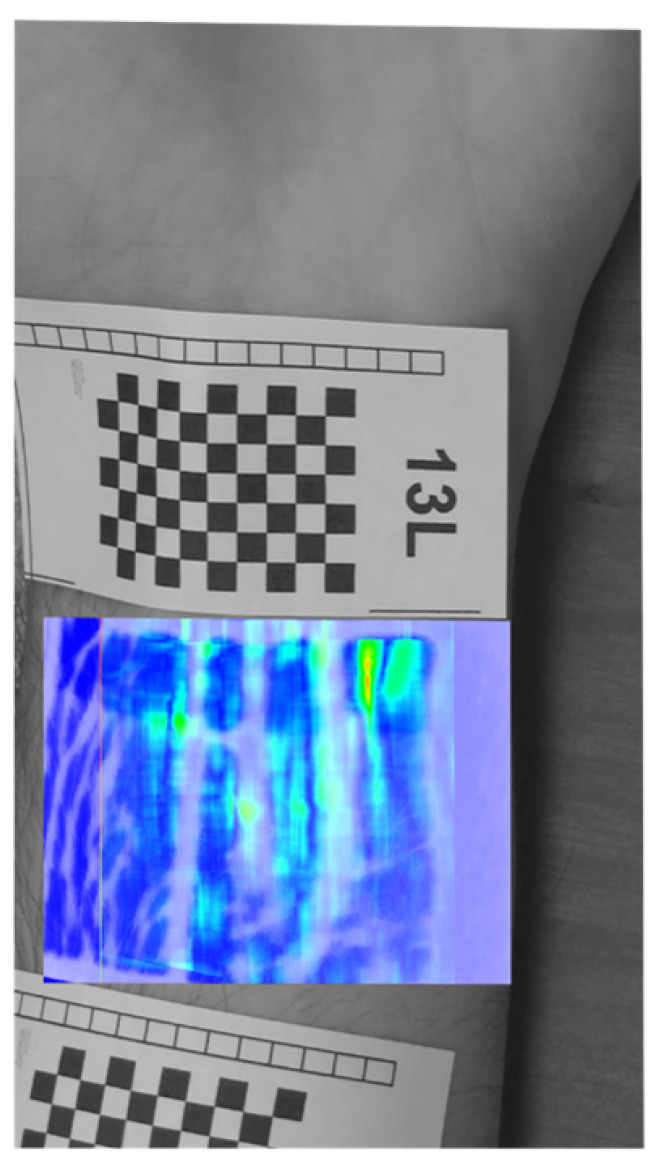
Cumulative shifts overlaid on the negative reference image.

**Figure 14:**
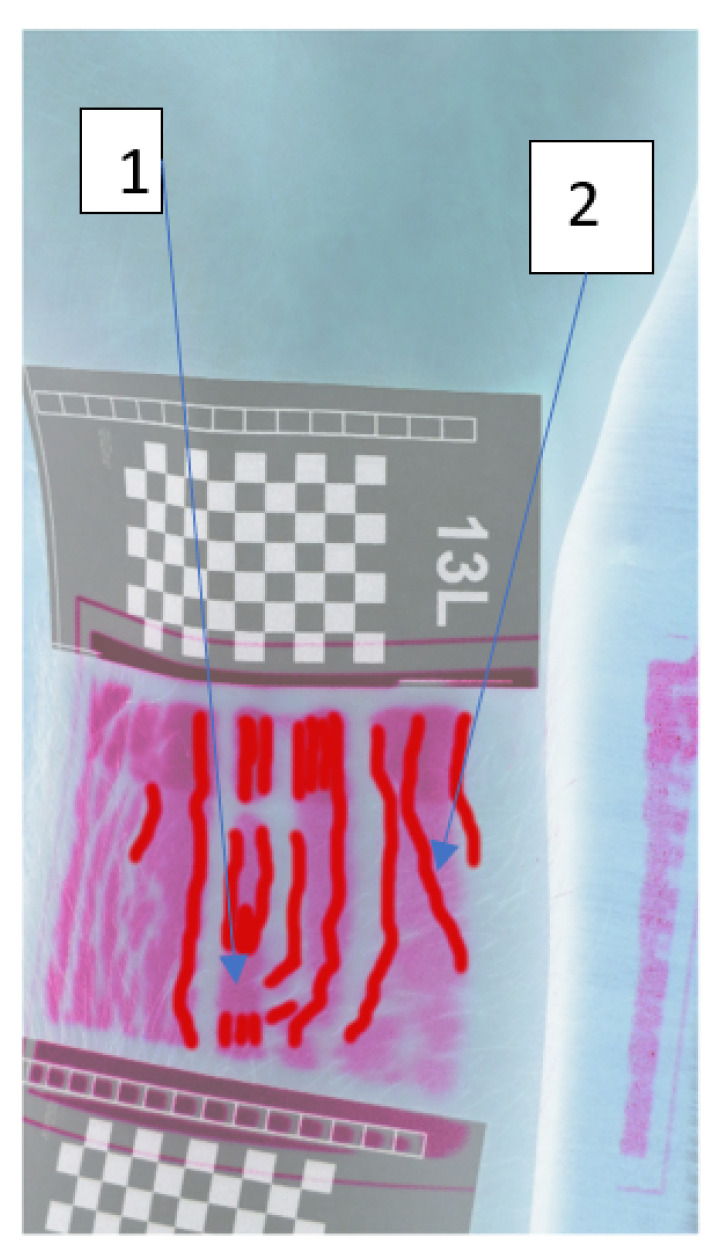
The manually traced edges of the veins detected.

Figure 12 shows cumulative (sum) shifts for 120 frames (movie with 30 FPS, that is, about 4 s of the movie) for 13L participant. Calculation parameters of the map was: a grid of points 400 × 310, the matching area for one point of the grid: 97 pixels. Dark blue places of the image indicate no shift, which means that they indicate the edges of the veins (where the skin does not pulsate). Figure 13 shows reference image with overlaid cumulative shifts from Figure 12. In our opinion, this is the least accurate and legible image of the veins, so based on this image, we created the manually traced edges of the veins detected—Figure 14. Some areas detected as ‘pulsating’ do not coincide with the base (1), and edges of veins not visible in the base were also detected (2).

The research carried out on a test group of 31 people confirmed that the proposed method can be used to detect pulsating veins, however, determining the qualitative parameters (accuracy of determining the location of veins, maximum depth of detected veins) of the method requires even more detailed tests. Our method allowed to detect the pulsation of about 70% of the veins visualized with the NAVI-60 device. Typically, compared to the reference device, the smallest veins were hardly visible. The calculations were made using our own software based on the OpenCV library. The average computation time for 120 measurement frames was 10 s (on the computer with Intel i5 8265 processor and 8GB RAM). According to the processor specifications and benchmark results, similar computation times should be possible with the Exynos 9820 processor used in the mobile device used for image acquisition. Therefore, it seems that the proposed algorithms can be fully implemented on mobile devices.

## 4. Discussion

The application of this method is to improve the patient’s quality of life during all types of medical procedures that were mentioned before. Considering the fact that there are a lot of these procedures performed daily it is highly important.

This method allows us to observe the thickness of the veins for a certain length. This allows an initial, quick determination of whether the patient should be referred for diagnostic tests for venous embolism and thrombosis. A completely different principle of operation compared to the competing NIR method allows for the visualization of subsequent phases of blood flow through the veins. In addition, unlike the NIR-based methods, the proposed method allows us to check if the blood flow in the veins is not slowed down or if there is a blood-returning disorder.

## 5. Conclusions

The above-described method allows the finding of veins. It is characterized by high accuracy and efficiency. However, despite many advantages, it is not an ideal method. One of the biggest issues of the proposed method is the fact that it depends on camera position. During the study, we noticed that during horizontal positioning of the hand relative to the camera, it failed to effectively visualize the vein. No problems were encountered when the hand was vertically positioned towards the camera. One of the biggest advantages of the proposed method is complete independence from specialized equipment; it can be performed with the use of an ordinary mobile phone using its camera and its computing power. The authors believe that this method is promising, so further optimization tests should be carried out.

## Data Availability

https://github.com/UTP-WTIiE/NovelMethodOfVeinDetection, accessed on 24 March 2021.

## References

[1] Timm U., McGrath D., Lewis E., Kraitl J., Ewald H. Sensor system for non-invasive optical hemoglobin determination. Proceedings of the SENSORS.

[2] Thom C.S., Dickson C.F., Gell D.A., Weiss M.J. (2013). Hemoglobin variants: Biochemical properties and clinical correlates. Cold Spring Harb. Perspect. Med..

[3] Lippi G., Baird G.S., Banfi G., Bölenius K., Cadamuro J., Church S., Cornes M.P., Dacey A., Guillon A., Hoffmann G. (2017). Improving quality in the preanalytical phase through innovation, on behalf of the European Federation for Clinical Chemistry and Laboratory Medicine (EFLM) Working Group for Preanalytical Phase (WG-PRE). Clin. Chem. Lab. Med..

[4] Giavarina D., Lippi G. (2017). Blood venous sample collection: Recommendations overview and a checklist to improve quality. Clin. Biochem..

[5] Lamperti M., Pittiruti M. (2013). II. Difficult Peripheral Veins: Turn on the Lights. Br. J. Anaest..

[6] Buowari O.Y. (2013). Complications of venepuncture. Adv. Biosci. Biotechnol..

[7] Miyake R.K., Zeman H.D., Duarte F.H., Kikuchi R., Ramacciotti E., Lovhoiden G., Vrancken C. (2006). Vein imaging: A new method of near infrared imaging, where a processed image is projected onto the skin for the enhancement of vein treatment. Dermatol. Surg..

[8] Juric S., Flis V., Debevc M., Holzinger A., Zalik B. (2014). Towards a low-cost mobile subcutaneous vein detection solution using near-infrared spectroscopy. Sci. World J..

[9] Tobisawa N., Namita T., Kato Y., Shimizu K. Injection assist system with surface and transillumination images. Proceedings of the 2011 5th International Conference on Bioinformatics and Biomedical Engineering.

[10] Wadhwani M., Sharma A.D., Pillai A., Pisal N., Bhowmick M. (2015). Vein detection system using infrared light. Int. J. Sci. Eng. Res..

[11] Lin C., Li M., Sun X. (2012). A finger vein recognition algorithm based on gradient correlation. Aasri Procedia.

[12] Peng J., Wang N., Abd El-Latif A.A., Li Q., Niu X. Finger-vein verification using Gabor filter and SIFT feature matching. Proceedings of the 2012 Eighth International Conference on Intelligent Information Hiding and Multimedia Signal Processing.

[13] Lutowski Z., Marciniak B., Marciniak T., Bujnowski S. (2017). Precision of sub-pixel image displacement measurements. J. Mach. Constr. Maint. Probl. Eksploat..

[14] Pan B., Li K. (2011). A fast digital image correlation method for deformation measurement. Opt. Lasers Eng..

[15] Khoo S.W., Karuppanan S., Tan C.S. (2016). A review of surface deformation and strain measurement using two-dimensional digital image correlation. Metrol. Meas. Syst..

